# Frailty, malnutrition, healthcare utilization, and mortality in patients with dementia and cognitive impairment obtained from hospital administrative data

**DOI:** 10.3389/fmed.2025.1540050

**Published:** 2025-02-26

**Authors:** Reshma Aziz Merchant, Ying Qiu Dong, Shikha Kumari, Diarmuid Murphy

**Affiliations:** ^1^Division of Geriatric Medicine, Department of Medicine, National University Hospital, Singapore, Singapore; ^2^Department of Medicine, Yong Loo Lin School of Medicine, National University of Singapore, Singapore, Singapore; ^3^The Value Office, National University Health System, Singapore, Singapore; ^4^Department of Orthopaedic Surgery, National University Hospital, Singapore, Singapore

**Keywords:** dementia, cost, length of stay, readmission, mortality, malnutrition

## Abstract

**Introduction:**

With aging populations, the prevalence of dementia, frailty and malnutrition will increase. The aim of this study is twofold (a) to determine the demographic data, including frailty and malnutrition prevalence in older patients with diagnosis of dementia and/or cognitive impairment and (b) to determine its impact on outcomes such as length of stay (LOS), readmission and mortality stratified by frailty status.

**Methods:**

Retrospective single-center cohort study conducted using hospital database on older patients ≥65 yrs. admitted to a tertiary hospital between March 2022 and Dec 2023 and discharged with either primary or secondary diagnosis of dementia or cognitive impairment. Data on age, gender, ethnicity, comorbidities, discharge diagnoses, Hospital Frailty Risk Score (HFRS), Clinical Frailty Scale (CFS), activity of daily living (ADL), 3-Minute Nutrition Screening and outcomes such as LOS, readmission, mortality and cost of hospitalization were extracted. Those aged between 65 to 74 years old were categorized as “young-old,” and ≥75 years old as “old-old.”

**Results:**

Dementia or cognitive impairment diagnosis was prevalent in 8.6% (3090) older patients, and 33.7% were malnourished. 54.5% were female with a mean age of 82.0 years. Almost one fourth were dependent on ADL. Based on frailty defined by (i) HFRS—26.0% had intermediate and 18.2% high frailty (ii) CFS—41.0% were mild/moderately frail, and 32.2% severely frail. Median LOS was 8 days. 30 and 90-days readmission rates were 23.2 and 35.4%, respectively. In-hospital mortality was 7.8% and 30-day mortality 14.0%. High HFRS (aOR 1.511, 95% CI: 1.089–2.097; *p* = 0.013), severe frailty (aOR 4.325, 95% CI: 0.960–2.684; *p* < 0.001) and terminal frailty (aOR 39.762, 95% CI: 18.311–86.344; *p* < 0.001) were significantly associated with inpatient mortality. Intermediate HFRS (aOR 1.682, 95% CI: 1.380–2.050; *p* < 0.001), mild/moderate frailty (1.609, 95% CI: 1.254–2.065; *p* < 0.01), high HFRS (aOR 2.178, 95% CI: 1.756–2.702; *p* < 0.001) and severe frailty (2.333, 95% CI: 1.804–3.017; *p* < 0.01) were significantly associated with 30-days readmission. The impact of malnutrition on healthcare utilization was highest in the old-old with high HFRS and severe frailty.

**Conclusion:**

Frailty and malnutrition have significant impact on healthcare utilization, readmission rates, and mortality among older adults with dementia and/or cognitive impairment.

## Introduction

With aging populations, the prevalence of dementia, frailty and associated disability will increase, putting strain on finite resources. Dementia encompasses a spectrum of neurological disorders characterized by progressive cognitive decline, impairment in activities of daily living, and mortality. The progression of dementia typically follows a gradual trajectory, starting with mild cognitive impairment and advancing to severe disability (1). This disease progression is intricately linked with frailty, a syndrome marked by decreased physiological reserve and increased vulnerability to stressors and malnutrition which is a risk factor for both frailty and dementia (2–4). Frailty is categorized into physical, social and cognitive frailty, and significantly increases risk of cognitive impairment and dementia (2, 5). The coexistence of dementia and frailty accelerates cognitive and physical decline, leading to increased healthcare utilization, disability, and mortality (6). Both prevention of dementia and frailty are global health priorities, and many countries are adopting population wide initiatives to delay the onset, reverse or delay the progression of these conditions (7, 8).

Singapore, one of the fastest-aging countries globally, projects that one in four of its population will be 65 years old and above by 2030, and nearly one in two by 2050 (9). A recent publication, studying the healthcare burden of cognitive impairment in the Singapore Chinese population, showed that these patients incurred 17.0% increased costs annually, mainly from emergency department visits and unplanned admissions (10). Data from the United Kingdom showed that the number of persons living with dementia (PLWD) who were hospitalized have increased by 93.0% between 2011 and 2017 (11). Similarly, the cost of their care during the same time period had doubled from £1.2bn to £2.7bn and costs due to emergency admissions were 30.0% higher than those with no dementia. This increase in admissions may be due to increased awareness and access to diagnostic facilities, longer life expectancies, increased multimorbidity, suboptimal integrated care or community resources at discharge, lack of caregiver availability and social isolation (9). Hospitalization is often harmful and distressing for older adults, more so for PLWD. The risks of functional decline, delirium, malnutrition, incontinence, nosocomial infections, falls, unnecessary tests and adverse drug events with prolonged length of stay (LOS) and readmissions are increased in this group of individuals (12, 13). Hospitalization has been associated with 1.7 to 3.3-fold increased decline in cognitive trajectory (14).

PLWD are at increased risk of malnutrition and prevalence varies depending on setting, stage of dementia and population being studied. Malnutrition rates may be as high as 47.8% in community dwelling to 75.6% in institutionalized PLWD with a pooled prevalence of 26.98% (15). This may be due to changes in taste and smell affecting appetite, poor oral health, physical limitation, forgetting to eat, psychosocial and behavioral factors and increase energy expenditure from wandering (16). Malnutrition is associated with poor quality of life, sarcopenia, falls, accelerated decline in cognition and frailty trajectory, hospitalization, and mortality (17). Hospitalization poses additional risks for PLWD such as delirium, prolonged bed rest, and nil by mouth orders (18, 19). The combined effects of dementia, frailty, and malnutrition create a vicious cycle that accelerates decline and increased mortality.

Previous studies have demonstrated that PLWD experience higher healthcare utilization and costs. However, there is a paucity of research on the impact of frailty and malnutrition in patients with dementia and/or cognitive impairment and its association with healthcare utilization, readmissions, mortality, and cost. Therefore, the aim of this study was twofold. Firstly, to determine the demographic data, including frailty status and malnutrition prevalence, in patients with diagnosis of dementia and/or cognitive impairment admitted to an academic hospital and secondly, to determine its impact on outcomes such as length of stay, readmission and mortality on this cohort, stratified by frailty status.

## Methodology

All patients over the age of 65 yrs (35,930 patients), admitted to a tertiary hospital from March 2022 to Dec 2023 were reviewed using the institution’s administrative database for diagnosis of dementia or cognitive impairment. Existing, de-identified data was extracted by executives from the Value Driven Outcome Department. Dementia or cognitive impairment diagnosis was based on either primary or secondary diagnosis. In addition, older patients were also included in the analysis if they were on any of the acetylcholinesterase inhibitors or memantine as a surrogate for diagnosis of dementia (Supplementary Table S1).

### Demographic data

Data on age, gender, ethnicity, underlying comorbidities, discharge diagnoses, age adjusted Charlson Comorbidity Index, Hospital Frailty Risk Score (HFRS), Clinical Frailty Scale (CFS) and activity of daily living (ADL) 2 weeks prior to admission and malnutrition was obtained from hospital administrative database. Those aged between 65 to 74 years old were categorized as young-old, and ≥ 75 years old as old-old.

Common discharge diagnoses were based on previous publications were such as delirium, pneumonia, urinary tract infection, constipation, hyponatremia, ischemic stroke, intracranial hemorrhage, acute myocardial infarction, congestive cardiac failure, orthostatic hypotension, osteoporotic fracture, Parkinson’s Disease, and sepsis (20). Malnutrition was defined using 3-Minute Nutrition Screening (3-MinNS) tool (score range between 0 and 9) (21). A score of ≥3 was used to diagnose malnutrition. ADL data was obtained 2 weeks prior to admission and accessed from nursing notes.

Information on premorbid CFS 2 weeks before admission was collected at the triage area in the emergency department for patients ≥65 years. CFS 1–4 were categorized under robust/vulnerable, 5–6 mild/moderate, 7–8 as severe frailty and 9 as terminally ill. HFRS scoring was computed using the ICD-10 codes and was initially described in 2018 by Gilbert et al. (22). It has since been validated in many continents and is associated with adverse outcomes such as mortality, and length of stay (23, 24). HFRS is classified into low <5, intermediate 5–15 and high >15. The Age-adjusted Charlson comorbidity index was initially validated to predict mortality is a constitute of weighted index of age, number and seriousness of comorbid disease (25).

### Outcome

Outcomes data such as LOS, readmission (within 30 and 90 days after discharge), in-hospital and 30-day mortality, and the cost of hospitalization was obtained from the hospital database. Cost was defined as the total cost of hospitalization to care provider per patient per episode during our observation window. Cost data was further categorized into laboratory, medication, radiology, occupational therapy, and physiotherapy cost.

### Statistical analysis

All data analysis was conducted on STATA Version 15. Frequencies and percentages were used to summarize the categorical variables. Continuous variables were presented with mean, standard deviation, median, and interquartile range (IQR). We adopted the t-test for continuous variables and the Pearson’s *χ*^2^ test for categorical variables to compare the statistical significance between young-old and old-old groups.

We stratified the patients into different groups according to their HFRS, CFS and malnutrition. Medians of the total cost and rates of readmission and in-hospital mortality were calculated for each group to compare the outcomes and costs across groups.

Logistic regressions were performed to estimate the odds ratio of HFRS and CFS on 30 and 90-day readmission, and in-hospital mortality. Multivariate linear regressions were conducted to estimate the effects of HFRS and CFS on total cost of hospitalization. The logarithm transformation was performed for the cost for normalization of the skewed distribution and proportional interpretation of results. We adjusted for both the demographics, including age, gender, ethnicity, and multimorbidity, in the regression analysis. The low frailty or robust/vulnerable group were used as reference groups.

### Ethics

The study was reviewed and approved by NUHS Research Office NUH-RNR-2024-0034. As anonymous data was obtained from the database, informed consent was not required.

## Results

### Demographics and prevalence of common comorbidities

There was a total of 3,090 older adults (≥65 years) diagnosed with dementia and/or cognitive impairment with 4,238 in-hospital admissions in this hospital between March 2022 and December 2023. Dementia or cognitive impairment diagnosis was prevalent in 8.6% of all older adults who were hospitalized, 54.5% were female with a mean age of 82.0 ± 8.0 years (Table 1). The top 3 diagnoses prevalent in almost one third of the patients were delirium (31.5%), pneumonia (33.3%), and UTI (32.5%). For comorbidity prevalence, hypertension (56.4%) was highest followed by diabetes mellitus (40.7%) and hyperlipidemia (40.5%). Based on frailty status defined by HFRS, 55.8% were classified as low, 26.0% as intermediate and 18.2% as high. Based on CFS, 25.1% were either robust or vulnerable, 41% had mild or moderate frailty, and 32.2% severe frailty. The prevalence of malnutrition was 33.7%.

**Table 1:** Demographics and outcome of dementia or cognitive impairment.

	All	65–74 years	≥ 75 years	*p*-Value
	3,090	643 (20.8)	2,447 (79.2)	
Demographics				
Gender				**<0.001**
Male	1,407 (45.5)	363 (56.5)	1,044 (42.7)	
Female	1,683 (54.5)	280 (43.6)	1,403 (57.3)	
Age (years)	82.0 ± 8.0	70.4 ± 2.7	85.1 ± 6.0	**<0.001**
Ethnicity				**<0.001**
Chinese	2,512 (81.3)	454 (70.6)	2058 (84.1)	
Malay	225 (7.3)	82 (12.8)	143 (5.8)	
Indian	181 (5.9)	54 (8.4)	127 (5.2)	
Others	172 (5.6)	53 (8.2)	119 (4.9)	
Discharge Diagnosis				
Delirium	973 (31.5)	138 (21.5)	835 (34.1)	**<0.001**
Pneumonia	1,028 (33.3)	177 (27.5)	851 (34.8)	**0.001**
UTI	1,004 (32.5)	180 (28.0)	824 (33.7)	**0.006**
Constipation	232 (7.5)	39 (6.1)	193 (7.9)	0.119
Hyponatremia	612 (19.8)	86 (13.4)	526 (21.5)	**<0.001**
Ischaemic Stroke	302 (9.8)	82 (12.8)	220 (9.0)	**0.004**
Intracranial Bleed	152 (4.9)	38 (5.9)	114 (4.7)	0.192
Acute Myocardial Infarction	373 (12.1)	63 (9.8)	310 (12.7)	**0.047**
Diabetes Mellitus	1,257 (40.7)	319 (49.6)	938 (38.3)	**<0.001**
Hypertension	1743 (56.4)	379 (58.9)	1,364 (55.7)	0.145
Hyperlipidaemia	1,252 (40.5)	307 (47.7)	945 (38.6)	**<0.001**
Heart failure	244 (7.9)	51 (7.9)	193 (7.9)	0.970
Orthostatic hypotension	263 (8.5)	57 (8.9)	206 (8.4)	0.718
Osteoporosis fractures	207 (6.7)	20 (3.1)	187 (7.6)	**<0.001**
Parkinson’s Disease	162 (5.2)	36 (5.6)	126 (5.2)	0.649
Sepsis	437 (14.1)	101 (15.7)	336 (13.7)	0.201
Hospital Frailty Risk Score				
Low	1721 (55.8)	375 (58.5)	1,346 (55.1)	**0.023**
Intermediate	801 (26.0)	173 (27.0)	628 (25.7)	
High	562 (18.2)	93 (14.5)	469 (19.2)	
Clinical Frailty Score^*^				**<0.001**
Robust (CFS 1–3)	321 (12.4)	115 (21.2)	206 (10.1)	
Vulnerable (CFS 4)	330 (12.7)	96 (17.7)	234 (11.4)	
Mild (CFS 5)	443 (17.1)	94 (17.3)	349 (17.1)	
Mod (CFS 6)	620 (23.9)	130 (23.9)	490 (23.9)	
Severe (CFS 7–8)	835 (32.2)	106 (19.5)	729 (35.6)	
Terminally ill (CFS 9)	41 (1.6)	2 (0.4)	39 (1.9)	
Age adjusted Charlson’s Comorbidity Index, median (IQR)	6 (IQR 4–7)	6 (IQR 4–7)	6 (IQR 4–8)	0.252
Malnutrition^^^	895 (33.7)	116 (20.7)	779 (37.2)	**<0.001**
Outcomes				
In-hospital Mortality	240 (7.8)	33 (5.1)	207 (8.5)	**0.005**
30-days Mortality	430 (13.9)	53 (8.2)	377 (15.4)	**<0.001**
30-days Readmission	718 (23.2)	148 (23.0)	570 (23.3)	0.883
90-days Readmission	1,093 (35.4)	229 (35.6)	864 (35.3)	0.885
Length of Stay since Admission (day)				
Mean	12.7 ± 15.2	15.9 ± 18.9	11.9 ± 14.0	**<0.001**
Median (IQR)	8 (IQR 4–16)	8 (IQR 5–19)	8 (IQR 4–15)	

*n*(%); *n* ± SD; malnutrition defined by 3 min Nutrition Screening score ≥ 3.

* Missing data *n* = 500.

^ Missing data for 441.Bold values indicate statistically significant differences between groups (*p* < 0.05).

**Figure 1:**
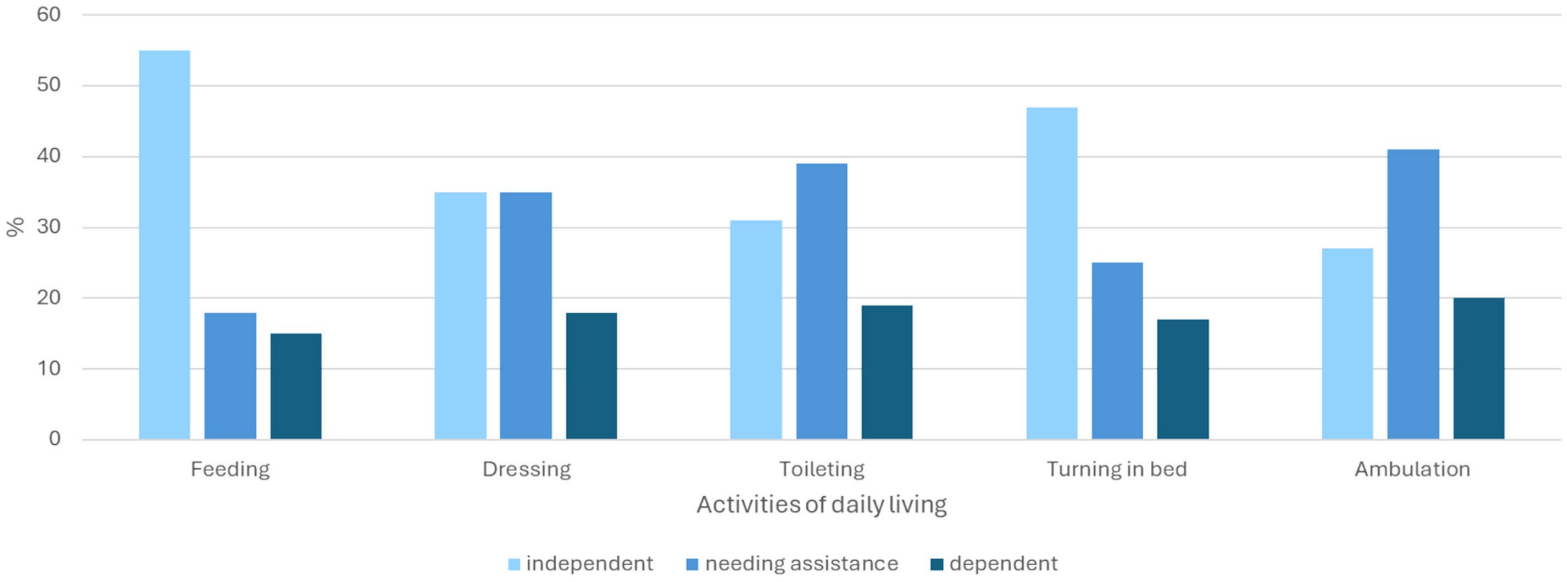
Activity of daily living 2 weeks prior to admission.

**Figure 2:**
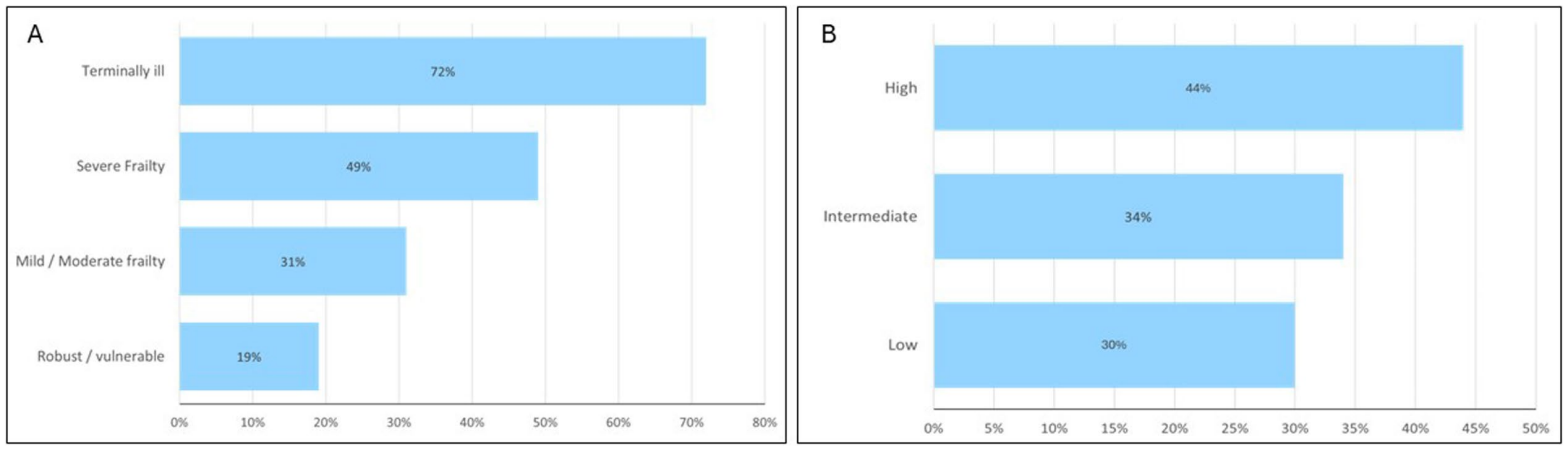
Prevalence of malnutrition across frailty groups defined by clinical frailty score **(A)** and hospital frailty risk score **(B)**.

There were significant differences in the demographics between the young-old compared with the old-old. There was higher prevalence of female in the old-old group (57.3% vs. 43.6%) and Chinese ethnic group (84.1% vs. 70.6%). With regards to discharge diagnoses, delirium (34.1%), pneumonia (34.7%) and UTI (33.7%) was more prevalent in the old-old. Similarly, other diagnoses more prevalent in the old-old include hyponatremia (21.5% vs. 13.4%), acute myocardial infarction (12.7% vs. 9.8%), and osteoporotic fracture (7.6% vs. 3.1%). The prevalence of ischemic stroke, diabetes mellitus and hyperlipidemia were lower in the old-old. Almost half of the young-old had underlying diabetes mellitus compared to one third of the old-old. Figure 1 shows that 15–20% of older patients with cognitive impairment were dependent on various activities of daily living prior to admission. Only one fourth were independent in ambulation. In the old-old compared to young-old, the prevalence of severe frailty based on CFS was almost double (35.6% vs. 19.5%) and high HFRS 25% higher (19.2% vs. 14.5%). Malnutrition was significantly higher in the old-old (37.2%) compared to young-old (20.7%). Half of those with severe frailty and more than two thirds of those with terminal frailty had underlying malnutrition (Figure 2). The prevalence was similarly high in those with high HFRS.

### Outcomes

**Figure 3:**
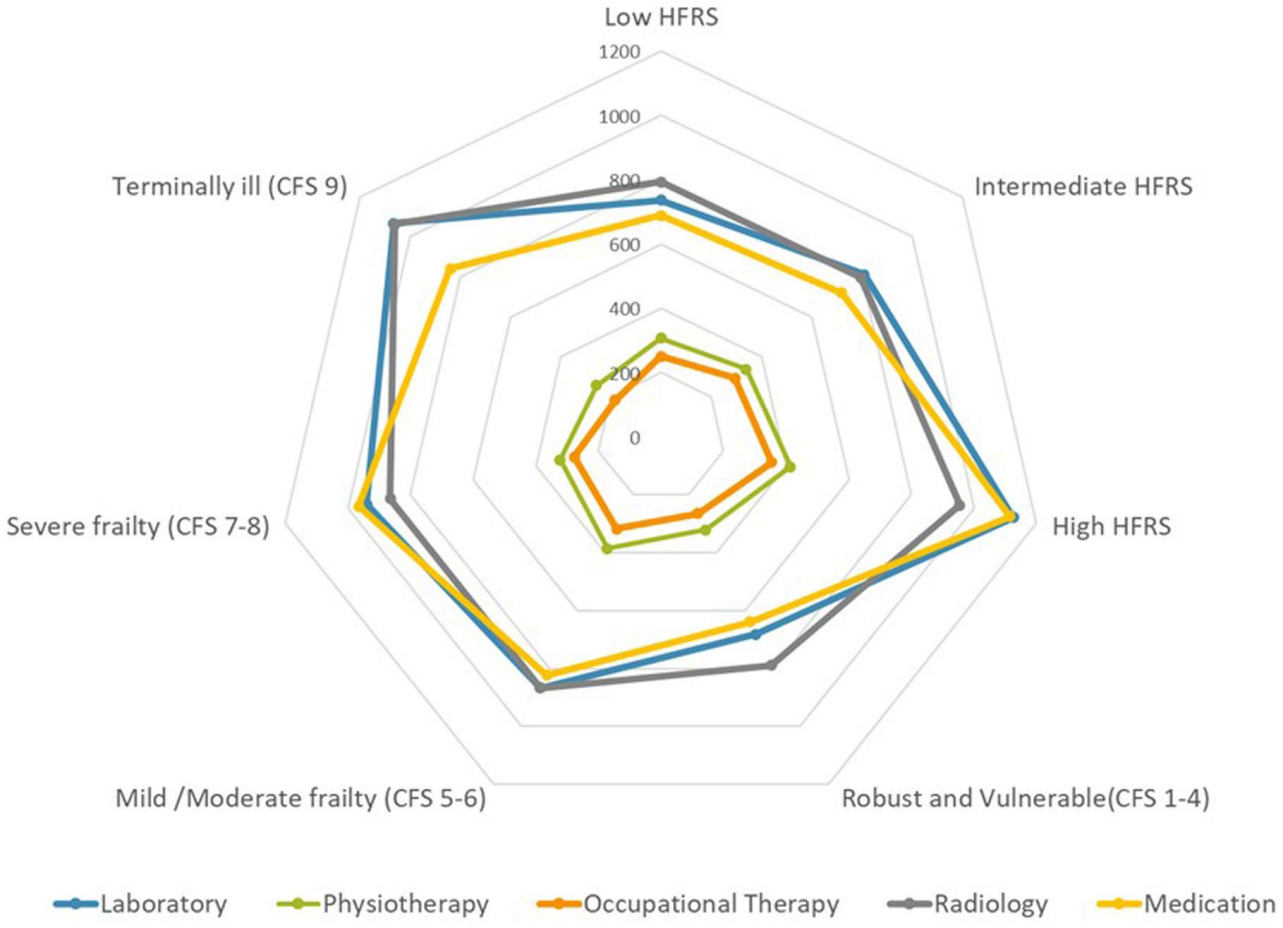
Categorical cost by hospital frailty risk score (HFRS) and clinical frailty scale (CFS).

**Figure 4:**
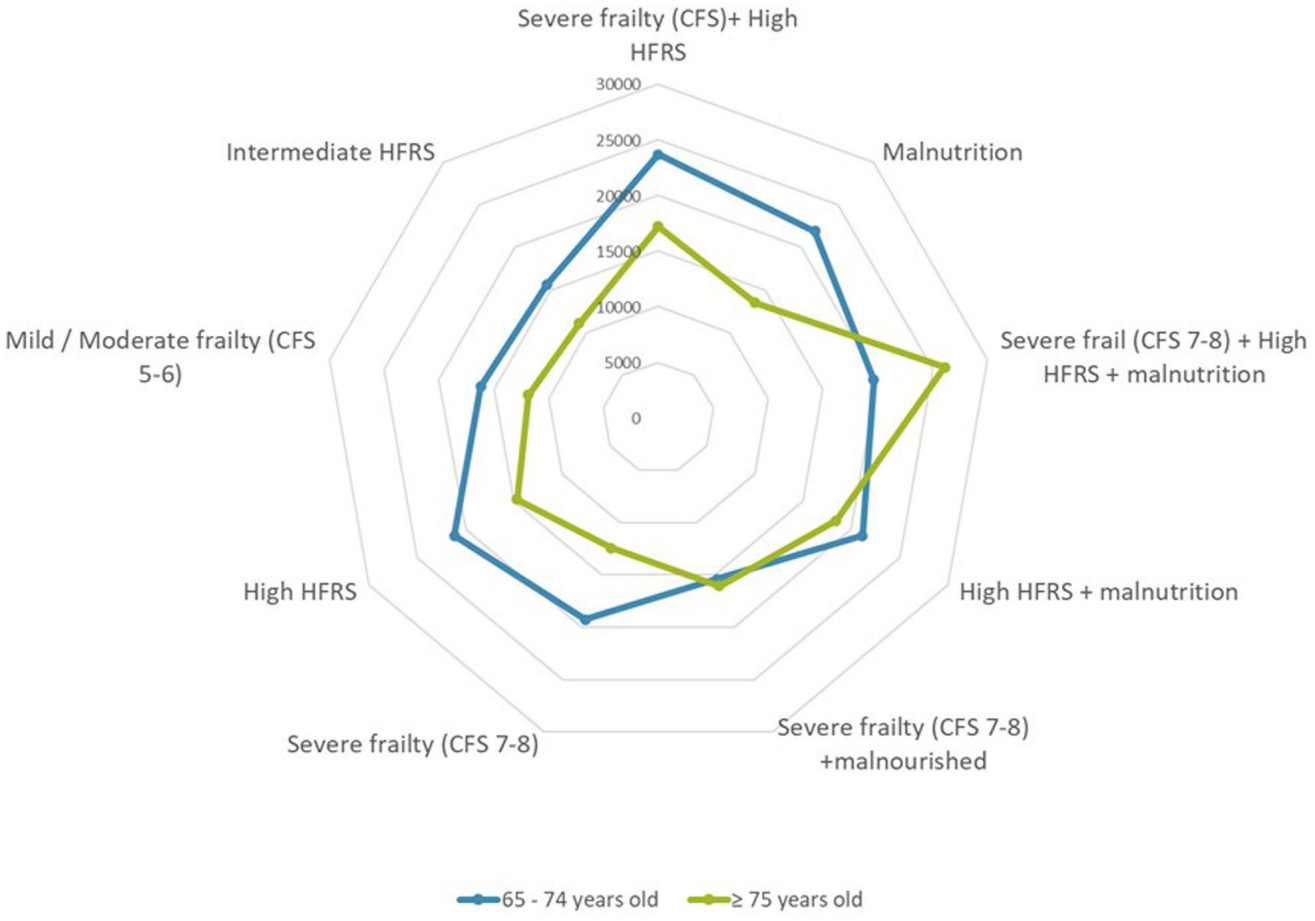
Cost breakdown by frailty status defined by hospital frailty risk score and/or clinical frailty scale in the presence or absence of malnutrition.

The overall median LOS was 8.0 days (IQR 8.0–16.0). Thirty-day and ninety-day readmission rates were 23.2 and 35.4%, respectively. In hospital mortality was 7.8% and 30-days mortality was 14.0%. Both the mean and median LOS were shorter in the old-old group. Indices for in-hospital mortality (8.5% vs. 5.2%), and 30 days mortality (15.4% vs. 8.2%) were significantly higher in the old-old compared with young-old. Breakdown of cost by frailty status is shown in Figure 3. Patients with severe frailty incurred higher costs in the laboratory, radiology, and/or medication category and high HFRS in the medication and laboratory category. Those terminally ill also incurred higher costs in the laboratory and radiology category. Median costs were significantly lower (by 25%) in the old-old than in the young-old across all frailty categories except those with high HFRS, severe frailty and malnutrition (Figure 4).

Tables 2, 3 shows outcomes by different frailty criteria with/without malnutrition in the young-old, and old-old. The results show that outcomes vary significantly with frailty levels, frailty screening tools or the presence of malnutrition. In the young-old group, the 90 days readmission rates among those with high HFRS, severe frailty, high HFRS + severe frailty, and high HFRS + severe frailty + malnutrition were 60.2, 44.3, 65.4 and 60.0%, respectively, (Table 2). In the old-old group, the 90 days readmission rates among those with high HFRS, severe frailty, high HFRS + severe frailty, and high HFRS + severe frailty + malnutrition were 48.6, 44.3, 55.7 and 61.7%, respectively. Patients classified as moderately frail + high HFRS similarly had high 90-days readmission rates (65.4% in the young-old and 58.3% in the old-old). Amongst those with malnutrition, 30- and 90-days’ readmission rates were between 41.8 and 43.9%. In the old-old terminally frail patients, the inpatient mortality rate was highest at 61.5%, followed by 17.5% amongst those with severe frailty + malnutrition (Table 3).

**Table 2:** Malnutrition, hospital frailty risk score, clinical frailty score, and readmission (30- and 90-day) in the young-old, and old-old.

	Number of patients (*n*)		Readmission (30D) *n*(%)		Readmission (90D) *n* (%)	
Age groups (years)	65–74	≥75	65–74	≥75	65–74	≥75
Hospital frailty risk score (HFRS)—based on ICD-10 Codes						
Intermediate	173	628	41 (23.7)	178 (28.3)	71 (41.0)	264 (42.0)
High	93	469	34 (36.6)	150 (32.0)	56 (60.2)	228 (48.6)
Clinical frailty scale (CFS)—based on mobility, balance, and activities of daily living						
Mild/Moderate frailty	224	839	62 (27.7)	198 (23.6)	92 (41.1)	315 (37.5)
Severe frailty	106	729	30 (28.3)	236 (32.4)	47 (44.3)	323 (44.3)
Terminal frailty	2	39	0 (0)	5 (12.8)	1 (50)	7 (18.0)
Hospital frailty risk score (HFRS) and clinical frailty score (CFS)						
Mild/Moderate frailty (CFS) + High HFRS	45	156	16 (35.6)	54 (34.6)	27 (60.0)	91 (58.3)
Severe frailty (CFS) + High HFRS	26	183	10 (38.5)	74 (40.4)	17 (65.4)	102 (55.7)
Mild/Moderate frailty (CFS) + Intermediate HFRS	59	217	17 (28.8)	63 (29.0)	25 (42.4)	90 (41.5)
Severe frailty (CFS) + Intermediate HFRS	28	193	7 (25.0)	71 (36.8)	11 (39.3)	98 (50.8)
Malnutrition, hospital frailty risk score and clinical frailty score						
Malnutrition	116	779	51 (43.9)	326 (41.8)	51 (43.9)	326 (41.8)
Severe frailty (CFS) + High HFRS + malnutrition	10	94	6 (60.0)	41 (43.6)	6 (60.0)	58 (61.7)
High HFRS + malnutrition	23	198	11 (47.8)	76 (38.4)	13 (56.5)	111 (56.1)
Severe frailty (CFS) + malnutrition	33	316	16 (48.5)	121 (38.3)	19 (57.6)	159 (50.5)

Malnutrition defined by 3 min Nutrition Screening score ≥ 3. Severe frailty: CFS score 7–8; Mild/Moderate frailty: CFS 5–6.

**Table 3:** Malnutrition, hospital frailty risk score, clinical frailty score, and in-hospital mortality in the young-old, and old-old.

	Number of patients (*n*)		In-hospital mortality *n* (%)	
Age groups (years)	65–74	≥75	65–74	≥75
Hospital frailty risk score (HFRS)—based on ICD-10 Codes				
Intermediate	173	628	6 (3.5)	52 (8.3)
High	93	469	10 (10.8)	51 (12.4)
Clinical frailty scale (CFS)—based on mobility, balance, and activities of daily living				
Mild/Moderate frailty	224	839	10 (4.5)	44 (5.2)
Severe frailty	106	729	13 (12.3)	105 (14.4)
Terminal frailty	2	39	1 (50)	24 (61.5)
Hospital frailty risk score (HFRS) and clinical frailty score (CFS)				
Mild/Moderate frailty (CFS) + High HFRS	45	156	4 (8.9)	11 (7.1)
Severe frailty (CFS) + High HFRS	26	183	4 (15.4)	25 (13.7)
Mild/Moderate frailty (CFS) + Intermediate HFRS	59	217	1 (1.7)	6 (2.8)
Severe frailty (CFS) + Intermediate HFRS	28	193	3 (10.7)	29 (15.0)
Malnutrition, hospital frailty risk score and clinical frailty score				
Malnutrition	116	779	9 (7.8)	104 (13.3)
Severe frailty (CFS) + High HFRS + malnutrition	10	94	0 (0)	13 (13.8)
High HFRS + malnutrition	23	198	2 (8.7)	29 (14.6)
Severe frailty (CFS) + malnutrition	33	316	3 (9.1)	55 (17.4)

Malnutrition defined by 3 min Nutrition Screening score ≥ 3. Severe frailty: CFS score 7–8; Mild/Moderate frailty: CFS 5–6.

Table 4 shows the association between different frailty screening tools (HFRS: Table 4A, CFS: Table 4B) and mortality, readmission, cost and LOS. Patients with higher or more severe frailty had considerably worse health outcomes compared with low frailty or robust/vulnerable group. High HFRS (aOR 1.511, 95% CI: 1.089–2.097; *p* = 0.013), severe frailty (aOR 4.325, 95% CI: 0.960–2.684; *p* < 0.001) and terminal frailty (aOR 39.762, 95% CI: 18.311–86.344; *p* < 0.001) were significantly associated with in-patient mortality. Intermediate HFRS (aOR 1.682, 95% CI: 1.380–2.050; *p* < 0.001), mild/moderate frailty (1.609, 95% CI: 1.254–2.065; *p* < 0.01), high HFRS (aOR 2.178, 95% CI: 1.756–2.702; *p* < 0.001) and severe frailty (2.333, 95% CI: 1.804–3.017; *p* < 0.01) were significantly associated with 30-days readmission. Similar findings were observed for readmissions within 90 days.

**Table 4:** Association of hospital frailty risk score (HFRS) (A), clinical frailty scale (CFS) (B) with readmission (30 and 90-days), in-hospital mortality, cost and length of stay.

A.			
Outcomes	Hospital Frailty Risk Score (Low HFRS as reference group)	Unadjusted OR (95% CI)	Adjusted OR (95% CI)
		*p* value	*p* value
In-hospital mortality	Intermediate	1.036 (0.749–1.433)	1.049 (0.756–1.455)
		0.831	0.776
	High	**1.616 (1.169–2.233)**	**1.511 (1.089–2.097)**
		**0.004**	**0.013**
Readmission within 30 days	Intermediate	**1.687 (1.384–2.055)**	**1.682 (1.380–2.050)**
		**<0.001**	**<0.001**
	High	**2.182 (1.761–2.704)**	**2.178 (1.756–2.702)**
		**<0.001**	**<0.001**
Readmission within 90 days	Intermediate	**1.900 (1.594–2.265)**	**1.892 (1.586–2.256)**
		**<0.001**	**<0.001**
	High	**2.700 (2.219–3.280)**	**2.709 (2.224–3.300)**
		**<0.001**	**<0.001**
		Coef. (95% CI)	Coef. (95% CI)
^*^Cost (Log transformation)	Intermediate	**0.135 (0.054–0.215)**	**0.122 (0.043–0.202)**
		**0.001**	**0.003**
	High	**0.370 (0.279–0.461)**	**0.398 (0.308–0.488)**
		**<0.001**	**<0.001**
^*^LOS (Log transformation)	Intermediate	**0.173 (0.091–0.255)**	**0.162 (0.081–0.243)**
		**<0.001**	**<0.001**
	High	**0.449 (0.356–0.543)**	**0.470 (0.377–0.562)**
		**<0.001**	**<0.001**

B.			
Outcomes	Clinical Frailty Scale (Robust/vulnerable as reference group)	Unadjusted OR (95% CI)	Adjusted OR (95% CI)
		*p* value	*p* value
In-hospital mortality	Mild/Moderate (CFS 5–6)	1.606 (0.960–2.684)	1.550 (0.925–2.599)
		0.071	0.096
	Severe (CFS 7–8)	**4.937 (3.066–7.950)**	**4.325 (2.666–7.016)**
		**<0.001**	**<0.001**
	Terminal (CFS 9)	**46.875 (21.848–100.573)**	**39.762 (18.311–86.344)**
		**<0.001**	**<0.001**
Readmission within 30 days	Mild/Moderate (CFS 5–6)	**1.558 (1.217–1.995)**	**1.609 (1.254–2.065)**
		**<0.001**	**<0.001**
	Severe (CFS 7–8)	**2.250 (1.752–2.889)**	**2.333 (1.804–3.017)**
		**<0.001**	**<0.001**
	Terminal (CFS 9)	0.668 (0.257–1.741)	0.688 (0.263–1.799)
		0.409	0.445
Readmission within 90 days	Mild/Moderate (CFS 5–6)	**1.551 (1.257–1.914)**	**1.583 (1.280–1.958)**
		**<0.001**	**<0.001**
	Severe (CFS 7–8)	**1.989 (1.599–2.474)**	**2.051 (1.639–2.567)**
		**<0.001**	**<0.001**
	Terminal (CFS 9)	0.606 (0.275–1.337)	0.620 (0.280–1.372)
		0.215	0.238
		Coef. (95% CI)	Coef. (95% CI)
^*^Total Cost (Log transformation)	Mild/Moderate (CFS 5–6)	**0.210 (0.117–0.304)**	**0.266 (0.173–0.358)**
		**<0.001**	**<0.001**
	Severe (CFS 7–8)	**0.266 (0.168–0.365)**	**0.368 (0.269–0.467)**
		**<0.001**	**<0.001**
	Terminal (CFS 9)	0.117 (−0.186–0.420)	0.230 (−0.068–0.529)
		0.450	0.131
^*^LOS (Log transformation)	Mild/Moderate (CFS 5–6)	**0.247 (0.152–0.343)**	**0.297 (0.202–0.392)**
		**<0.001**	**<0.001**
	Severe (CFS 7–8)	**0.345 (0.245–0.445)**	**0.433 (0.332–0.534)**
		**<0.001**	**<0.001**
	Terminal (CFS 9)	0.167 (−0.141–0.476)	0.262 (−0.043–0.568)
		0.287	0.092

Adjusted for age, gender, and ethnicity.

* Multivariate linear regression using logarithm transformation.Bold values indicate statistically significant differences between groups (*p* < 0.05).

The cost of care for patients with intermediate HFRS was 12.2% (*p* = 0.003) and high HFRS 39.8% (*p* < 0.01) higher than those without frailty (Table 4A). Patients with intermediate HFRS had 16.2% (*p* < 0.001) longer length of stay while those with high HFRS had 47% (*p* < 0.001) longer length of stay (Table 4A). The findings were similar for frailty based on CFS scores where patients with mild/moderate frailty incurred 26.6% (*p* < 0.001) and severe frailty 36.8% (*p* < 0.001) more cost than the robust/vulnerable group. Patients with mild/moderate frailty and severe frailty had 29.7% (*p* < 0.001) and 43.3% longer length of stay, respectively, compared to the robust/vulnerable frailty group (Table 4B).

Table 5 shows association of high HFRS, severe frailty and malnutrition in combination with readmission (30 and 90-days), in-hospital mortality, cost and LOS. Reference was made to the absence of malnutrition. High HFRS, severe frailty + malnutrition incurred 47.3% (*p* = 0.001) higher cost and 51.3% longer LOS. High HFRS+ malnutrition and severe frailty+malnutrition were associated with increased inpatient mortality (aOR 1.906, 95% CI: 1.070–3.394; *p* = 0.029 and aOR 2.149, 95% CI: 1.391–3.322; *p* = 0.001 respectively).

**Table 5:** Association of high Hospital Frailty Risk Score (HFRS), severe frailty and malnutrition with readmission (30 and 90-days), in-hospital mortality, cost and length of stay.

	Outcome	Unadjusted OR (95% CI)	Adjusted OR (95% CI)
		*p* value	*p* value
Severe frailty (CFS) + High HFRS + malnutrition (reference: severe frailty + high HFRS)^*^	In-hospital mortality	0.795 (0.361–1.747)	0.725 (0.323–1.630)
		0.568	0.436
	Readmission within 30 days	1.515 (0.869–2.643)	1.618 (0.916–2.858)
		0.143	0.097
	Readmission within 90 days	1.455 (0.839–2.522)	1.513 (0.865–2.645)
		0.182	0.147
		Coef. (95% CI)	Coef. (95% CI)
	^*^Total Cost (Log transformation)	**0.441 (0.169–0.713)**	**0.473 (0.204–0.742)**
		**0.002**	**0.001**
	^*^LOS (Log transformation)	**0.486 (0.201–0.770)**	**0.513 (0.232–0.795)**
		**0.001**	**0.000**

	Outcome	Unadjusted OR (95% CI)	Adjusted OR (95% CI)
High HFRS + malnutrition (reference: high HFRS)	In-hospital mortality	**2.142 (1.220–3.760)**	**1.906 (1.070–3.394)**
		**0.008**	**0.029**
	Readmission within 30 days	1.269 (0.891–1.808)	1.390 (0.963–2.007)
		0.186	0.079
	Readmission within 90 days	0.985 (0.708–1.371)	1.078 (0.765–1.520)
		0.929	0.666
		Coef. (95% CI)	Coef. (95% CI)
	^*^Total Cost (Log transformation)	−0.023 (−0.187–0.142)	0.068 (−0.095–0.232)
		0.785	0.413
	^*^LOS (Log transformation)	0.018 (−0.152–0.187)	0.105 (−0.065–0.274)
		0.839	0.226

	Outcome	Unadjusted OR (95% CI)	Adjusted OR (95% CI)
Severe frailty (CFS) + malnutrition [reference: severe frail (CFS)]	In-hospital mortality	**2.209 (1.440–3.389)**	**2.149 (1.391–3.322)**
		**0.000**	**0.001**
	Readmission within 30 days	1.341 (0.997–1.804)	1.326 (0.981–1.791)
		0.053	0.066
	Readmission within 90 days	1.132 (0.860–1.492)	1.137 (0.858–1.505)
		0.377	0.371
		Coef. (95% CI)	Coef. (95% CI)
	^*^Total Cost (Log transformation)	−0.110 (−0.244–0.023)	−0.048 (−0.181–0.085)
		0.105	0.478
	^*^LOS (Log transformation)	−0.102 (−0.239–0.035)	−0.048 (−0.185–0.089)
		0.144	0.493

Adjusted for age, gender, and ethnicity.

* Multivariate linear regression using logarithm transformation.Bold values indicate statistically significant differences between groups (*p* < 0.05).

**Figure 5:**
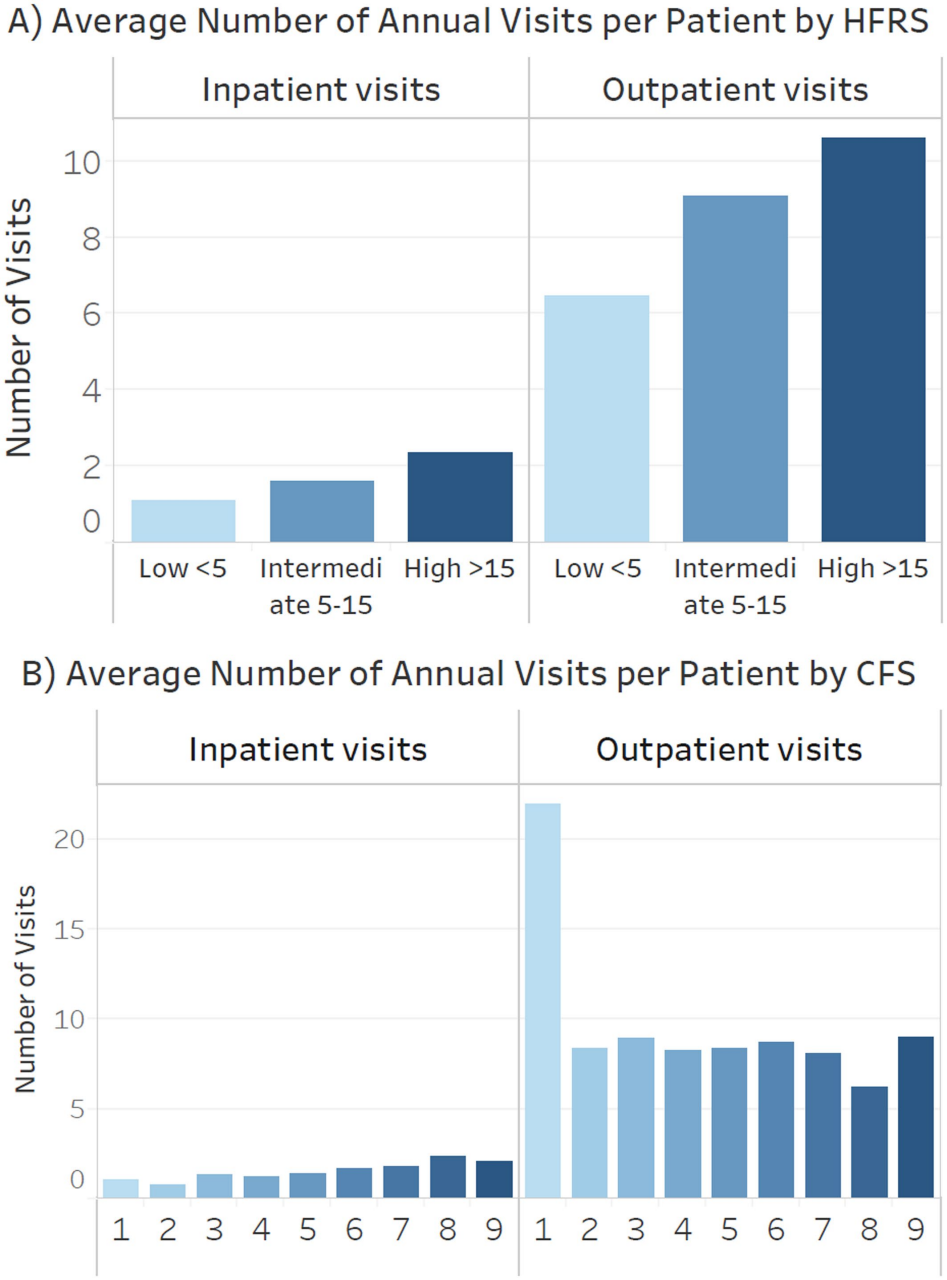
Annual inpatient admissions and outpatient visits by **(A)** Hospital Frailty Risk Score (HFRS) **(B)** clinical frailty scale (CFS).

Figure 5 (A: HFRS, B: CFS) shows the number of inpatient and outpatient visits by different frailty categories. Patients with high HFRS had an average of 11, terminally ill 9 and severe frailty 6 annual outpatient visits. Patients with high HFRS, severe frailty or terminally ill had an average of 2.5 annual inpatient admissions (Figure 5).

## Discussion

Patients with dementia and/or cognitive impairment constitute a heterogeneous group. They are at higher risk of hospitalization hazards, increased healthcare utilization and mortality (26). However, studies on the impact of frailty and malnutrition in this population are limited. Our results demonstrated that frailty, irrespective of the screening tools used, was significantly associated with a higher likelihood of in-patient mortality, 30-day or 90-day readmission, and increased healthcare utilization, including LOS and costs, among patients with cognitive impairment and/or dementia. Although there was no significant difference in the 30- and 90-day readmissions between young-old and old-old, the former incurred higher costs and exhibited longer LOS. Inpatient, and 30-days mortality was significantly higher in the old-old. The presence of malnutrition further exacerbated these outcomes, highlighting the compounded negative impact of both malnutrition and frailty on healthcare utilization and prognosis. Malnutrition in patients with severe frailty or high HFRS was associated with increased in-patient mortality. In patients who had both high HFRS and severe frailty, malnutrition was associated with increased cost and LOS.

The prevalence of dementia and/or cognitive impairment was 8.6%, and almost 80% were ≥75 years old. The prevalence of dementia and cognitive impairment is known to increase with age and is often underdiagnosed. Depending on age groups and specialty, more than one third of older patients ≥70 years old may have underlying dementia but diagnosis in the acute setting is often complicated by concurrent presence of delirium where many of these patients may also have underlying dementia (27, 28). Despite including both cognitive impairment and/or dementia, the prevalence in our study population was relatively low. Other surrogates for cognitive impairment such as impairment in ADL could serve as a useful surrogate for frailty and cognitive impairment (29). However, another study using hospital database similarly found prevalence of dementia in 7.5% of hospitalized older patients (30). The top three discharge diagnoses were delirium, pneumonia and urinary tract infection. Pneumonia incidence is more frequent in patients with dementia, and accounts for 25% of hospitalization (30, 31). These patients are at an increased risk of recurrent pneumonia and increased mortality (30, 31). One third of our old-old participants had a discharge diagnosis of delirium. Previous studies showed that delirium may be prevalent in almost half of hospitalized older patients with dementia (32).

One third of the patients had severe frailty defined by CFS but only one fifth had high HFRS. HFRS is derived from ICD-10 codes whereas CFS better reflects function, ADL and mobility (22, 33). The combination of both screening tools which measured different aspects with malnutrition in our study population was associated with increased LOS and cost, but either one with malnutrition, only with inpatient mortality. At least mild/moderate frailty based on CFS was prevalent in three quarters of hospitalized patients with dementia and/or cognitive impairment. The prevalence of frailty in patients with dementia in the acute care setting is reported to be between 50.8 and 91.8% (34). The cost and LOS were significantly higher in the frail patients. Unlike other studies which showed that old age was associated with greater healthcare utilization, our study showed that young-old cohort with intermediate or mild/moderate and high or severe frailty incurred higher costs and longer LOS (35, 36). Besides increased inpatient and 30-days mortality in the old-old, other reasons for shorter LOS and lower cost may be that a large proportion of patients aged 75 years or older in this institution are cared for by geriatricians. A prior study showed that patients under geriatricians’ care had lower LOS and incurred reduced costs (20). An observational study from US acute hospitals, where the mean age of study participants was 82.5 years, reported a significant association of patients with a comorbid diagnosis of dementia with longer LOS, higher mortality, but lower costs and fewer procedures (37). This was attributed to communication issues, less intense care and administrative delays.

More than one third of the old-old in our study population were malnourished. Hospitalized patients are usually complex with high morbidity. In addition, PLWD often experience difficulties in meal preparation, have higher prevalence of anorexia of aging, swallowing difficulties, polypharmacy, mobility issues or access to food (38). Our findings are similar to a recent meta-analysis by Arifin et al. which reported a prevalence of 32.52% and a further 46.80% at risk of malnutrition (4). Borda et al. similarly reported a prevalence of 28.7% amongst those with mild dementia, and greater functional decline over 5 years in those who were malnourished (3). Malnutrition is a well-recognized modifiable risk factor for dementia, frailty, disability and mortality. It is associated with reduced quality of life, sarcopenia, increased morbidity, and accelerated decline in both cognition and frailty (3, 17, 39, 40). While most older adults get screened for malnutrition in the hospital and long-term care setting (16), they may not be routinely screened in primary care or memory clinic. Good practice guidelines on dementia should incorporate nutrition screening in older patients at every healthcare encounter (16). The 2024 ESPEN guideline on nutrition and hydration in dementia recommends routine screening for malnutrition and dehydration, elimination of potential causes, oral nutritional supplement to improve nutrition status, and adequate social support (38).

Diagnosis of dementia is associated with double the risk of mortality (30). The prevalence of inpatient mortality in patients with dementia and/or cognitive impairment was 7.8%, which was significantly lower than other studies which showed prevalence as high as 24.3% (30). However, the old-old patients in our study with terminal illness had an in-patient mortality rate of 61.5, and 17.4% in those with severe frailty and malnutrition. Patients with severe frailty had four times higher odds, and those with terminal illness had forty times higher odds for in-patient mortality. The presence of malnutrition in those with high HFRS or severe frailty was associated with a 2-fold increase in the odds of in-patient mortality compared with those without malnutrition.

The readmissions rates, especially in the mild/moderate to severe frailty and/or high HFRS and malnourished old-old group were increased where up to two-thirds were readmitted at 90 days and slightly more than one third at 30 days. Both high HFRS and severe frailty were associated with more than double the risk of 30- and 90-days readmission, with high HFRS showing the greatest risk of 90 days readmission. The association was also significant for mild/moderate or intermediate frailty. Almost three quarters of our study population were assisted or dependent on ambulation, feeding and toileting prior to hospital admission. Our findings are in keeping with a systematic review by Ma et al. comprising of 19 studies which showed 30-day readmission rates were between 7 to 35% in persons living with dementia (41). Management of patients with dementia and/or cognitive impairment is complex due to concurrent presence of multimorbidity, functional consequences, behavioral problems, polypharmacy, food refusal and increasing dementia severity (36, 42–44). In addition, this group of patients are particularly susceptible to delays in diagnosis and management, hazards of hospitalization and possible even ageism (45). PLWD who are hospitalized may often feel isolated, may be subjected to repetitive monitoring and endless tests, barrier in communication or sensory impairment may lead to emotional trauma, lack of sleep and restraints may further accelerate functional decline and delirium risk (12).

Although we know that ‘Less is More’ in patients with advanced dementia or frail, the medications, laboratory and radiology cost were higher in the high HFRS, and severe frailty group (33, 46). With increasing severity of frailty, outpatient visits did not reduce and those with terminal illness still had 9 visits/year. The young-old incurred higher costs. Another study from the same institution showed that healthcare utilization was significantly higher in the young-old group in the last 12 months of their life (47). Yorganci et al. showed that both rates and LOS of unplanned hospital admissions are higher in the last 12 months of their life (48). Documentation of advanced dementia in the clinical notes has also shown to be associated with shorter LOS, lower use of intensive care unit and 30-day mortality (49). Other good practices include improving the uptake of advanced care planning and deprescribing drugs with lowest benefit to harm ratio through STOPP&START or Beers criteria (50, 51).

Studies have shown that despite initiatives to improve healthcare professionals’ knowledge and other healthcare initiatives in managing patients with dementia, barriers to optimal care delivery in this group persists due to competing priorities and emphasis on managing chronic diseases, social isolation, lack of adequate care transition including caregiver education and handover, fragmentation of care and lack of multidisciplinary collaboration (52, 53). In multi-ethnic countries like Singapore, language barrier, social determinant of health and cultural differences may also have an impact on healthcare utilization. A recent narrative review by Browne et al. which included 16 studies from the USA, Taiwan, Australia, Canada, Sweden, Japan, Denmark and The Netherlands showed that factors such as reduced mobility, increased numbers of chronic conditions, inadequate discharge panning and interdisciplinary collaboration, socio-economic inequalities amongst different ethnic groups and behavioral symptoms increased readmission rates (54). It is believed that 20–40% of these admissions could be avoided.

While the strength of our study includes a robust database, with comprehensive data on nutrition, healthcare utilization and frailty, there are several limitations which warrants mention. First, the accuracy of data obtained is subject to accuracy of diagnosis, documentation and coding. The prevalence of dementia and/or cognitive impairment was much lower in our study population. However, this may not undermine our findings on the association of frailty with increased healthcare utilization, and mortality. Second, we had no information on other factors which may impact healthcare utilization and mortality such as severity of illness, advance care planning (ACP), social determinants of health, caregiver education, discharge destination and community resources. ACP has been associated with reduced healthcare utilization and LOS (55). In addition, while we had no information on staging of dementia, but dependent in ADL and frailty could serve as a surrogate measure. One third of these patients were classified under severe frailty category who may be approaching end of life, where discussion on goal-directed care and appropriate care could have an impact on overall outcome. Third, we have no information on intervention and compliance with nutritional supplements which could have had an impact on the outcome. Last, CFS is based on records obtained at the emergency department triage. While emergency department healthcare professionals have been trained to record premorbid CFS 2 weeks prior to admission, there may be a recall bias in the setting of acute illness or there may not be any caregivers available. CFS is able to measure the dynamic nature of frailty whereas HFRS is a measure of comorbidities and may not change with change in functional status. Combining both with malnutrition was associated with worst outcomes in patients with dementia and/or cognitive impairment.

Our study highlights that health administration data is a crucial resource for determining healthcare quality and outcomes. This study provides essential insights for policymakers and healthcare providers responsible for establishing standards for future care of patients with dementia and cognitive impairment, frailty and malnutrition. Implementing health system approaches like the Age-Friendly Health System’s 4 M’s (What Matters, Mobility, Medication, and Mentation) (35), including enhancing health care professionals knowledge on person centered care for dementia and conducive environment design should be a priority in every healthcare institution (53). Integration of frailty and nutrition assessments in healthcare encounters, alongside comprehensive geriatric assessment and consideration of social determinants of health, discharge destinations, caregiver education, and seamless care transitions, is vital for managing readmissions, healthcare utilization, and mortality rates (56, 57). By adopting a goal-directed care approach, we can better address the needs of this vulnerable population and improve their overall health outcomes, indirectly enhancing healthcare utilization.

Assessment and management of patients with dementia require a multifaceted approach which includes early detection of frailty, malnutrition and other geriatric syndromes, and these individuals will benefit from comprehensive geriatric assessment and targeted interventions. Future research should focus on exploring the impact of advanced care planning, social determinants of health, malnutrition intervention such as oral nutrition solution, caregiver education, discharge planning, and care transition on readmission, healthcare utilization, and mortality in these patients. This will further clarify the factors influencing these outcomes and inform strategies to better manage care for individuals with dementia and cognitive impairment.

## Conclusion

This study underscores the significant impact of frailty, and malnutrition in patients with dementia and/or cognitive impairment on healthcare utilization, readmission rates, and mortality. These findings highlight the essential need for healthcare providers to prioritize assessments of frailty and nutrition in patients with dementia or cognitive impairment to better manage their health outcomes. Given the severe vulnerability of this population segment, addressing these issues through regular frailty assessments and integrating data from administrative records can lead to more informed care decisions and resource allocation.

## Data Availability

The datasets presented in this article are not readily available because data on cost will not be available for sharing. Requests to access the datasets should be directed to mdcram@nus.edu.sg.
