# Supplementary material for: Frailty, malnutrition, healthcare utilization, and mortality in patients with dementia and cognitive impairment obtained from hospital administrative data
Source: Front Med (Lausanne). 2025 Feb 26;12:1540050. doi: 10.3389/fmed.2025.1540050 (PMC11897001; doi:10.3389/fmed.2025.1540050)
Supplement: Supplementary file 1 [file Table_1.docx]

**Supplementary Table 1. Codes for dementia and cognitive impairment**

| **Category** | **Code** | **Description** | **Tag** |
| --- | --- | --- | --- |
| **Service Code** | 00002010X1 | Donepezil HCL 10mg tablet | Dementia |
|  | 00002011X3 | Donepezil HCL 5mg tablet | Dementia |
|  | 000412035E | DONEPEZIL HCL 5MG TABLET (ARICEPT) | Dementia |
|  | 000412057F | DONEPEZIL 10MG TAB (ARICEPT) | Dementia |
|  | 000412093E | DONEPEZIL ORODISPERSIBLE 5MG TAB | Dementia |
|  | 000412094D | DONEPEZIL ORODISPERSIBLE 10MG TAB | Dementia |
|  | 000412096W | DONEPEZIL 23MG TABLET (ARICEPT) | Dementia |
|  | 2010X1 | DONEPEZIL HCL 10MG TABLET | Dementia |
|  | 2011X3 | DONEPEZIL HCL 5MG TABLET | Dementia |
|  | MAF_DRUG54 | Donepezil Hydrochloride | Dementia |
|  | 000412037L | RIVASTIGMINE 1.5MG CAPSULE (EXELON) | Dementia |
|  | 000412038J | RIVASTIGMINE 3MG CAPSULE (EXELON) | Dementia |
|  | 000412039H | RIVASTIGMINE 4.5MG CAPSULE (EXELON) | Dementia |
|  | 000412040L | RIVASTIGMINE 6MG CAPSULE (EXELON) | Dementia |
|  | 000512016I | RIVASTIGMINE 4.6MG/24H PATCH (EXELON) | Dementia |
|  | 000512017H | RIVASTIGMINE 9.5MG/24H PATCH (EXELON) | Dementia |
|  | 000512018G | RIVASTIGMINE 13.3MG/24H PATCH (EXELON) | Dementia |
|  | MAF_DRUG70 | RIVASTIGMINE | Dementia |
|  | 000412058D | GALANTAMINE 12MG TABLET (REMINYL) | Dementia |
|  | 000412059W | GALANTAMINE 4MG TABLET (REMINYL) | Dementia |
|  | 000412060F | GALANTAMINE 8MG TABLET (REMINYL) | Dementia |
|  | 000412082G | GALANTAMINE HBR 8MG PR CAP (REMINYL PR) | Dementia |
|  | 000412083F | GALANTAMINE HBR 16MG PR CAP (REMINYL PR) | Dementia |
|  | 000412084E | GALANTAMINE HBR 24MG PR CAP (REMINYL) | Dementia |
|  | 000428149I | MEMANTINE HCL 10MG TABLET (EBIXA) | Dementia |
|  | 000428428G | MEMANTINE 10MG TAB | Dementia |
| **ICD Code** | F000 | F000 Dementia in Alzheimer's disease with early onset | Dementia |
|  | F001 | F001 Dementia in Alzheimer's disease with late onset | Dementia |
|  | F002 | F002 Dementia in Alzheimer's disease, atypical or mixed type | Dementia |
|  | F009 | F009 Dementia in Alzheimer's disease, unspecified | Dementia |
|  | F010 | F010 Vascular dementia of acute onset | Dementia |
|  | F011 | F011 Multi-infarct dementia | Dementia |
|  | F012 | F012 Subcortical vascular dementia | Dementia |
|  | F013 | F013 Mixed cortical and subcortical vascular dementia | Dementia |
|  | F018 | F018 Other vascular dementia | Dementia |
|  | F019 | F019 Vascular dementia, unspecified | Dementia |
|  | F020 | F020 Dementia in Pick's disease | Dementia |
|  | F021 | F021 Dementia in Creutzfeldt-Jakob disease | Dementia |
|  | F022 | F022 Dementia in Huntington's disease | Dementia |
|  | F023 | F023 Dementia in Parkinson's disease | Dementia |
|  | F024 | F024 Dementia in human immunodeficiency virus [HIV] disease | Dementia |
|  | F028 | F028 Dementia in other specified diseases classified elsewhere | Dementia |
|  | F03 | F03 Unspecified dementia | Dementia |
|  | G300 | G300 Alzheimer's disease with early onset | Dementia |
|  | G301 | G301 Alzheimer's disease with late onset | Dementia |
|  | G308 | G308 Other Alzheimer's disease | Dementia |
|  | G309 | G309 Alzheimer's disease, unspecified | Dementia |
|  | G311 | G311 Senile degeneration of brain, not elsewhere classified | Dementia |
|  | G310 | G310 Circumscribed brain atrophy | Dementia |
|  | R54 | R54 Senility | Dementia |
|  | R418 | R418 Other and unspecified symptoms and signs involving cognitive functions and awareness | Cognitive Impairment |
|  | G318 | G318 Other specified degenerative diseases of nervous system | Cognitive Impairment |
|  | F067 | F067 Mild cognitive disorder | Cognitive Impairment |
